# Low-Cost, High-Yield ZnO Nanostars Synthesis for Pseudocapacitor Applications

**DOI:** 10.3390/nano12152588

**Published:** 2022-07-28

**Authors:** Gisella Maria Di Mari, Giacometta Mineo, Giorgia Franzò, Salvatore Mirabella, Elena Bruno, Vincenzina Strano

**Affiliations:** 1 Dipartimento di Fisica e Astronomia “Ettore Majorana”, Università degli Studi di Catania, Via S. Sofia 64, 95123 Catania, Italy; gisella.dimari@dfa.unict.it (G.M.D.M.); giacometta.mineo@dfa.unict.it (G.M.); elena.bruno@dfa.unict.it (E.B.); 2 Consiglio Nazionale delle Ricerche, Istituto per la Microelettronica e i Microsistemi (CNR-IMM), Università degli Studi di Catania, Via S. Sofia 64, 95123 Catania, Italy; giorgia.franzo@ct.infn.it (G.F.); vincenzina.strano@ct.infn.it (V.S.)

**Keywords:** zinc oxide nanostars, pseudocapacitor, oxygen vacancies, substrate contribution evaluation, neutral pH

## Abstract

Energy storage devices based on earth-abundant materials are key steps towards portable and sustainable technologies used in daily life. Pseudocapacitive devices, combining high power and high energy density features, are widely required, and transition metal oxides represent promising building materials owing to their excellent stability, abundance, and ease of synthesis. Here, we report an original ZnO-based nanostructure, named nanostars (NSs), obtained at high yields by chemical bath deposition (CBD) and applied as pseudocapacitors. The ZnO NSs appeared as bundles of crystalline ZnO nanostrips (30 nm thin and up to 12 µm long) with a six-point star shape, self-assembled onto a plane. X-ray diffraction (XRD), scanning electron microscopy (SEM), and photoluminescence spectroscopy (PL) were used to confirm the crystal structure, shape, and defect-mediated radiation. The ZnO NSs, dispersed onto graphene paper, were tested for energy storage by cyclic voltammetry (CV) and galvanostatic charge–discharge (GCD) analyses, showing a clear pseudocapacitor behavior. The energy storage mechanism was analyzed and related to oxygen vacancy defects at the surface. A proper evaluation of the charge stored on the ZnO NSs and the substrate allowed us to investigate the storage efficiency, measuring a maximum specific capacitance of 94 ${F\;g}^{-1}$ due to ZnO nanostars alone, with a marked diffusion-limited behavior. The obtained results demonstrate the promising efficacy of ZnO-based NSs as sustainable materials for pseudocapacitors.

## 1. Introduction

Over the past decades, electrical energy consumption has recorded the fastest growth relative to total energy demand [1], boosted by the most recent and contingent economic and climatic conditions, with electricity being essential in different fields such as industry, the residential sector, services, and commercial sectors [2,3]. With the expected global energy demand increase and the need to expand the availability of renewable sources through the transition to low-carbon electricity systems [4], developing sustainable, efficient, and clean energy storage technologies has become one of the required approaches for the material science communities worldwide [5].

Energy storage devices, such as batteries, electric double-layer capacitors (EDLCs), and pseudocapacitors, play the leading role in these fields. Batteries, storing the energy via a bulk redox reaction, are most suitable for applications with high energy density [6,7]. By contrast, in EDLCs, the electrochemical storage arises from charges electrostatically stored at the electrode interface [8,9], and they have high power density [10,11,12]. Pseudocapacitive materials combine the advantages of EDLCs and batteries since capacitive and charge-transfer processes coexist [13,14,15].

A proper device design development, involving economical and eco-friendly production processes, represents the most promising approach for succeeding in high-performance supercapacitors; in particular, nanomaterial-based electrodes play a crucial role in electrochemical energy storage [10] since the higher surface area can significantly contribute to improving the specific capacitance [16].

In recent years, transition metal oxides have been widely applied for electrochemical capacitors with high power density, taking advantage of their pseudocapacitive behavior. These oxides provide higher specific capacitance values because of their many oxidation states, characteristic of pseudocapacitive materials [17].

Among transition metal oxides, zinc oxide, an inorganic semiconductor relatively abundant in nature, has been extensively studied as an anode material candidate for lithium-ion batteries [18]. Owing to its eco-friendly nature and good electrochemical activity, ZnO also became a promising electrode material for supercapacitors [19,20,21]. Moreover, ZnO can be easily nanostructured in a multitude of morphologies by employing many different methods [22,23,24,25,26]. Solution routes for ZnO nanostructure synthesis have a multitude of advantages, such as cost containment, a simple laboratory setup, low-temperature processes, and fast kinetics growth [27].

It is worth mentioning that ZnO's properties are morphology dependent, which makes this material even more attractive for several scientific purposes [28,29], including energy storage applications.

Jayachandiran et al. reported ZnO nanoparticle fabrication obtained using a sol-gel approach, following a rGO/ZnO composite fabrication, obtained using ultrasonic-assisted solution-phase synthesis [30]. The composite, coated on nickel foil, showed pseudocapacitive behavior and a specific capacitance value of 312 ${F\;g}^{-1}$ at 5 ${\mathrm{mV}\;s}^{-1}$. Luo et al. synthesized ZnO tetrapods, prepared using vapor transport methods, coated onto Ni foam [31]. The material showed pseudocapacitive behavior, with a specific capacitance value of 160 ${F\;g}^{-1}$ obtained at 1 ${A\;g}^{-1}$. Finally, Guo et al. obtained ZnO nanoparticles using a hydrothermal method, following a graphene–ZnO composite formation using microwave treatment [32]. Again, the material exhibited a pseudocapacitive behavior, with an obtained specific capacitance of 201 ${F\;g}^{-1}$ at 1 ${A\;g}^{-1}$. All of these function in a neutral condition by using 1 M ${\mathrm{Na}}_{2}{\mathrm{SO}}_{4}$, where corrosion problems related to water splitting reactions are avoided [33]. Nonetheless, in these papers, the substrate’s role was not considered nor evaluated, while it is known that C or Ni compounds act as energy storage materials. To optimize the study of the effective energy storage process in ZnO nanostructures, a proper substrate contribution evaluation is needed. This exercise is also useful in order to compare different ZnO nanostructure data in the literature, which usually appear as deposited on different substrates.

In this paper, a new nanostar-like ZnO nanostructure obtained at a very large yield by means of chemical bath deposition (CBD) is presented. These nanostars are composed of many crystalline wires, highly ordered within a common plane. The as-prepared nanostars were used as supercapacitor active material, and the energy storage mechanism was correlated with structural and morphological features.

## 2. Materials and Methods

### 2.1. Synthesis of ZnO Nanostars

ZnO nanostars (NSs) were synthesized by means of chemical bath deposition (CBD). Starting from an aqueous solution of zinc nitrate and hexamethylenetetramine (HMTA) [34], the ZnO NSs production was attained by adding ammonium fluoride to the bath [26]. Three solutions (50 mL each) were separately prepared with deionized water (MilliQ, 18 MΩ cm): (i) 25 mM zinc nitrate hexahydrate ($\mathrm{Zn}{\left({\mathrm{NO}}_{3}\right)}_{2}\cdot 6{\;H}_{2}O$, purum p.a., crystallized, ≥99.5%, Sigma Aldrich, Milan, Italy); (ii) 25 mM HMTA (${\left({\mathrm{CH}}_{2}\right)}_{6}N_{4}$ ≥ 99.5%, Sigma Aldrich, Milan, Italy); and (iii) 16 mM ammonium fluoride (${\mathrm{NH}}_{4}F$, ≥99.99%, Sigma-Aldrich, Milan, Italy). These solutions were placed in a bain-marie configuration to reach and maintain the desired temperature of 90 °C. Once the thermal equilibrium was reached, the three solutions were mixed in a large beaker where the 90 °C temperature was still maintained. $\mathrm{Zn}{\left({\mathrm{NO}}_{3}\right)}_{2}$ and the HMTA solutions were mixed first, then after few seconds, the ${\mathrm{NH}}_{4}F$ solution was added. In order to study the growth kinetics, the synthesis solution was sampled at different growth times (0.5, 1, 3, 6, 10, 20, and 30 min). After 30 min, the solution was removed from the bain-marie configuration and left to reach room temperature.

The obtained solution was then washed with deionized water 4 times by means of decantation. The nanostar powders were finally dried in an oven in vacuum at 100 °C for 16 h (hereafter simply called NSs). A part of the product was then annealed in air onto a hot plate at 300 °C for one hour (hereafter called AnnNSs).

### 2.2. Characterization

The NS surface morphology was analyzed by using a Scanning Electron Microscope (Gemini field emission SEM Carl Zeiss SUPRA 25, Carl Zeiss Microscopy GmbH, Jena, Germany) equipped with an EDAX PV7715/89-ME energy-dispersive X-ray (EDX) spectrometer for elemental characterization. SEM images were analyzed by using ImageJ software to improve the brightness and contrast [35].

The ZnO NS crystal structure was examined by X-ray diffraction (XRD) using a Rigaku Smartlab diffractometer (Rigaku, Tokyo, Japan), equipped with a rotating anode of Cu $K_{\alpha }$ radiation operating at 45 kV and 200 mA in the grazing incidence mode (0.5°).

Photoluminescence (PL) measurements were performed by pumping with the 325 nm (3.81 eV) line of a He–Cd laser chopped through an acousto-optic modulator at a frequency of 55 Hz. The PL signal was analyzed using a single grating monochromator, detected with a Hamamatsu visible photomultiplier, and recorded with a lock-in amplifier using the acousto-optic modulator frequency as a reference [36].

Electrochemical measurements were carried out at room temperature using a VersaSTAT4 potentiostat (Princeton Applied Research, Oak Ridge, TN, USA), and a three-electrode setup was used with a platinum wire as a counter electrode, a saturated calomel electrode (SCE) as a reference electrode, and the ZnO NS samples on graphene paper (GP) substrates (240 µm thick, Sigma Aldrich, St. Louis, MO, USA) as a working electrode (WE). Solutions of 1 M ${\mathrm{Na}}_{2}{\mathrm{SO}}_{4}$, 1 M NaCl, and 1 M KCl (Sigma Aldrich, St. Louis, MO, USA, ≥85%) were used as supporting electrolytes. Cyclic voltammetry (CV) and galvanostatic charge–discharge (GCD) analyses were conducted in the potential range 0÷0.6 V vs. SCE (${\mathrm{Na}}_{2}{\mathrm{SO}}_{4}$), −0.1÷0.5 V (NaCl) and −0.2÷0.4 V (KCl). Electrochemical impedance spectroscopy (EIS) analyses were conducted at 0.3 V vs. SCE (in an ${\mathrm{Na}}_{2}{\mathrm{SO}}_{4}$ solution with a frequency range of 0.1–10,000 Hz and an amplitude of 10 mV RMS).

## 3. Results and Discussion

### 3.1. Material Characterization

**Figure 1:**
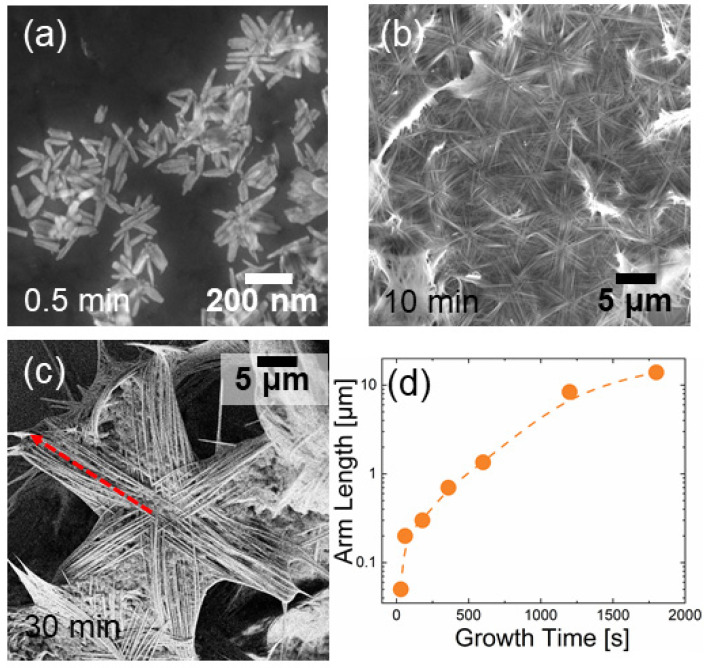
Kinetic study of ZnO NS growth. SEM images of nanostructures sampled after a growth of 0.5 (**a**), 10 (**b**), and 30 min (**c**); distribution of arm length as a function of time (**d**). The dashed red line in (**c**) indicates the arm length of an NS.

The zinc oxide nanostructure morphology can be seen in Figure 1a–c, which show SEM images of NSs dropped on Si substrates after different CBD durations. Beyond rare single wires, most of the precipitate appeared in the form of nanostars, with each one made of six coplanar arms starting from a common center. Such arms are composed of a bunch of parallel wires, and each star draws six equally spaced angles. It is worth noting that the ZnO wurtzite phase has a hexagonal symmetry, which could explain the nanostructures’ peculiar shape. As the CBD time increased, the arm length and thickness also increased.

A cross-linked center can be recognized, from which every wire starts with a lateral width of 70 nm, reaching a dimension of 20–30 nm on the tip (Figure S1). Once annealed, the morphology did not appreciably change (Figure S2).

Figure 1a–c show the SEM micrographs of NSs grown for 0.5, 10, and 30 min, respectively. After just half a minute, the stars’ central part was developed, while the arms clearly began to arrange themselves in preferential directions. After 30 min, the nanostars were very extended; their arms appeared dense with wires as long as 10–12 μm (dashed red line).

Arm length evolution as a function of time is plotted in Figure 1d. There was an initial explosive growth kinetics within 10 min, but the growth rate decreased at longer growth times.

**Figure 2:**
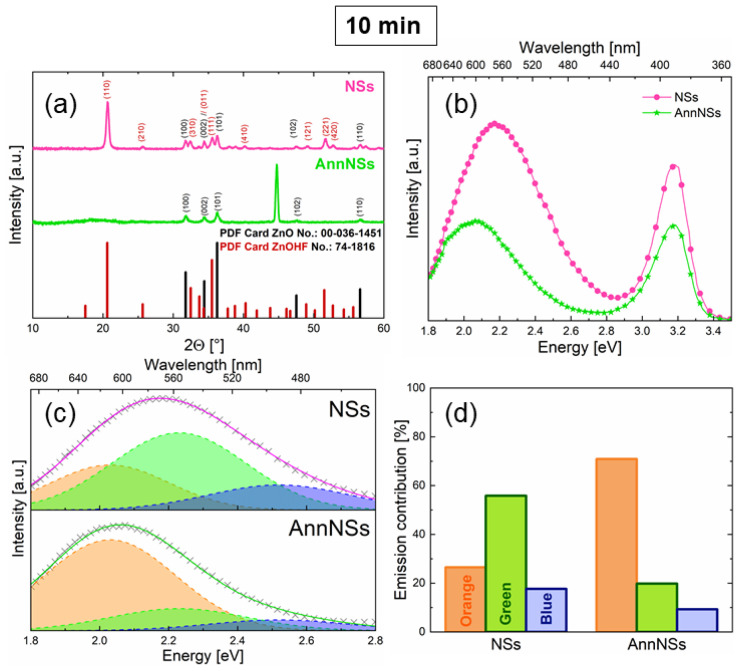
(**a**) XRD pattern of NSs and AnnNSs grown for 10 min; (**b**) room-temperature photoluminescence spectra; (**c**) visible emission band fitting with blue, green, and orange contributions, and (**d**) histogram of fit contributions for both NSs and AnnNSs.

Figure 2a shows the XRD patterns of 10-min-grown NSs before and after the annealing process. The NSs sample already shows many crystallographic peaks, which recall the hexagonal ZnO peaks at 2θ = 31.77°, 34.42°, 36.25°, 47.54°, and 56.60° corresponding to (100), (002), (101), (102), and (110), respectively (see PDF Card No. 00-036-1451 in Figure 2a). In addition, there is a sharp peak at 20°, more intense than the other crystallographic peaks, due to the ZnOHF presence in the powders (PDF Card No. 74-1816 in Figure 2a) that is also responsible for (100) and (101)’s peculiar peaks splitting into two components [37]. Once annealed, this hydroxy fluoride species almost disappeared, producing pure zinc oxide powders. The 45° peak was related to the carbon tape used as substrate for AnnNSs [38]. The co-existence of the ZnO and ZnOHF phase in the NS powder was also confirmed by EDX analyses, as shown in Figures S3 and S4, revealing the presence of oxygen, zinc, and fluorine in the NSs. The PL spectra were acquired for both NSs and AnnNSs, as shown in Figure 2b. All the emission spectra were composed of a UV peak (2.7–3.5 eV) and a broad visible band (1.8–2.7 eV). Although the profile of the emission spectra is characteristic of ZnO [39], a small contribution of the ZnOHF phase in the near-UV–visible region [40,41] cannot be entirely excluded. At first glance, annealing in air induced a redshift and a visible band reduction. As the visible band is typically associated with defects [39], it can be concluded that thermal annealing reduces these defects. In order to better understand this aspect, the visible PL was analyzed in detail.

The visible emission band can be described as a convolution of three Gaussian components attributed to the Blue (B, 2.52 eV), Green (G, 2.23 eV), and Orange (O, 2.03 eV) states [39].

In Figure 2c, the visible bands of the NSs and AnnNSs samples were fitted with the above Gaussian components, with FWHM fixed at 0.4 eV. The histograms in Figure 2d show the percent contribution of B, G, and O emissions for the NSs and AnnNSs. The B, G, and O emissions are related to radiation mediated by states into the ZnO bandgap [29,39]. The B emission is related to zinc vacancies ($V_{\mathrm{Zn}}^{2-}$) states; the G emission is related to singly ionized oxygen vacancies $(V_{O}^{+}$) states at the surface; while the O emission is associated with oxygen vacancies at the ZnO core.

The annealing process led to a strong G band reduction and a great O contribution enhancement, as also confirmed by the clear redshift observed in Figure 2b. It is plausible to think that thermal annealing in air supplies oxygen atoms that recombine O vacancies at the surface, thus significantly reducing the G emission. Such surface defect modifications will have some effects on energy storage performance, as detailed below.

Grown nanostars are a ZnO and ZnOHF mixed phase, which converts to pure ZnO after annealing in air. A large amount of surface defects was present in the as-grown samples; such defects were recombined by air annealing at 300 °C. The ease of synthesis can be a great advantage for scalability; however, some nanostar yield estimations still needed to be conducted. The nanostar synthesis yield was evaluated by assuming the whole process to be limited by a Zn reactant and by considering a 1:1 molar relationship between zinc nitrate and zinc oxide, as expected. The NS yield was obtained by weighting the amount of NSs, grown for 10 min, after the annealing process (where only ZnO was present) in comparison to the utilized Zn nitrate amount. The ratio between ZnO and Zn nitrate moles indicated a nanostar yield as high as 40%. As reported in the literature [42], the yield of solution-derived ZnO nanopowders strongly depends on pH value, with higher yields achieved in highly basic media. NSs are synthesized in solutions with a pH value of 5.7; therefore, a 40% yield may be considered promising for high-volume production.

### 3.2. Electrochemical Measurements

**Figure 3:**
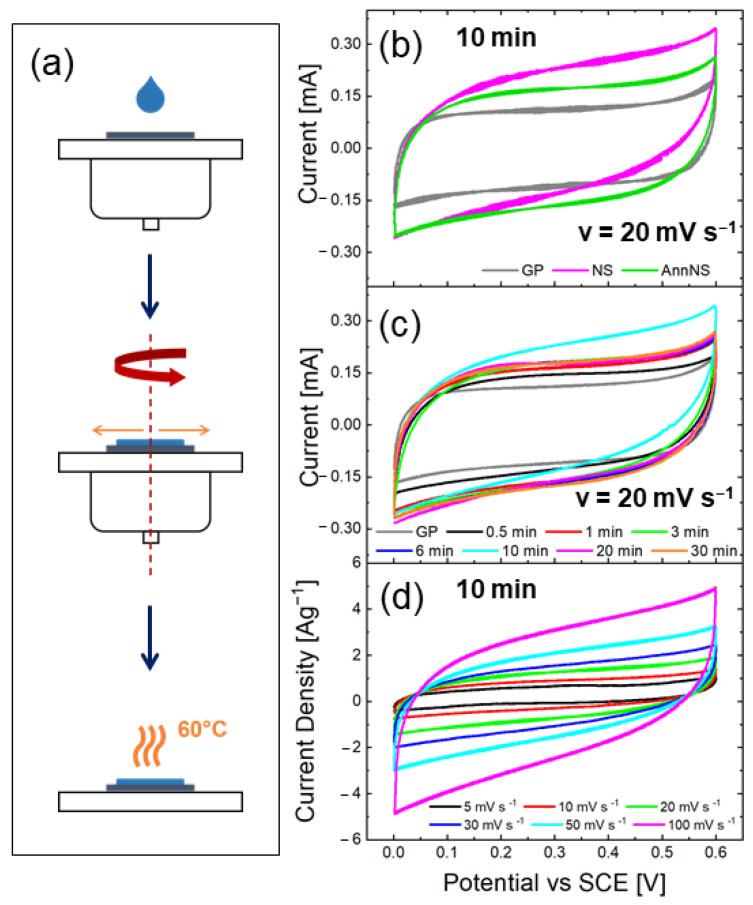
(**a**) Schematic of sample preparation for electrochemical characterization. CV curves in 1 M ${\mathrm{Na}}_{2}{\mathrm{SO}}_{4}$ of GP substrate (grey line); nanostars as prepared (magenta line) and after annealing (green line) at 20 mV/s (**b**); CV curves in 1 M ${\mathrm{Na}}_{2}{\mathrm{SO}}_{4}$ of NSs with 0.5, 1, 3, 6, 10, 20, and 30 min growth times and GP at 20 mV/s (**c**); CV curves in 1 M ${\mathrm{Na}}_{2}{\mathrm{SO}}_{4}$ of as-prepared NS at different scan rates (**d**).

To evaluate the ZnO NS electrochemical performances, CV and GCD analyses were performed on ZnO NSs-based electrodes, using a three-electrode setup. The WE was obtained by spin coating (300 rpm, 5 min) 40 μL of an NSs or AnnNSs aqueous solution (concentration of 2 mg mL^−1^) on a $1\;{\mathrm{cm}}^{2}$ graphene paper substrate, as shown in Figure 3a. The electrode was then dried on a hot plate at 60 °C in air, obtaining a mass of 0.2 mg, measured with a Mettler Toledo (Columbus, OH, USA) MX5 Microbalance (sensitivity: 0.01 mg). It should be noted that particular care was taken in order to have electrodes with the same mass so as to easily compare the electrochemical performances.

Figure 3b shows the CVs of 10 min ZnO NSs as grown and after annealing, compared with bare graphene paper (GP). As expected, the bare substrate was active in the charge storage process with a CV typical of an electric double-layer capacitor (EDLC) [15]. Such activity of GP in charge storage was taken into account to investigate the ZnO NS net ability in storing charges.

When the GP was covered with as-grown ZnO NSs, a larger CV area was recorded, almost double in size, highlighting a significant pseudocapacitive storage mechanism attributable to ZnO NSs. It is worth noting that the substrate mass was some orders of magnitude greater than the ZnO NSs mass (0.2 mg); thus, a specific capacitance comparison is meaningless; however, for a solid evaluation of ZnO NS energy storage efficiency, the substrate contribution must be evaluated. The annealed ZnO NSs showed a smaller charge storage ability in comparison to the as-grown one. Indeed, the PL analysis evidenced that annealing induced a reduction of oxygen vacancy defects, which are expected to contribute to the storage mechanism through surface adsorption processes [29].

Moreover, the XRD results showed that the as-grown NSs partly contained a ZnOHF phase whose layered structure could facilitate charged ion intercalation [43].

In Figure 3c, CV curves for NSs with different growth times are presented, again compared to the GP curve, at a chosen scan rate of $\nu \;=20{\;\mathrm{mV}\;s}^{-1}$. The bare GP substrate always showed a lower charge storage ability in comparison to that of GP covered with ZnO NSs, regardless of the ZnO NS growth times. By comparing GP covered with ZnO NSs at different growth times, what emerged is that, by increasing arm length, the stored charge increased up to 10 min, whereas for longer growth times, the charge storage started to decrease.

Figure 3d shows CV curves acquired for NSs grown for 10 min in 1M ${\mathrm{Na}}_{2}{\mathrm{SO}}_{4}$ at different scan rates, from 5 to 100 ${\mathrm{mV}\;s}^{-1}$, which are useful to further deepen the charge storage mechanism.

The stored charge (Q_c_, mC) can be determined from the CV curves as follows [44]:

$$Q_{c}=\frac{\int IdV}{\upsilon }$$

where *V* is the applied potential (V), *I* is the measured current (mA), and *υ* is the scan rate voltage $\left({V\;s}^{-1}\right)$. Consequently, the specific capacitance (C_s_, ${F\;g}^{-1}$) can be determined from the CV curves as follows [45]:

$$C_{s}=\frac{\int IdV}{m\upsilon \Delta V}$$

where *m* is the active NS mass (mg), and Δ*V* is the potential range (V).

**Figure 4:**
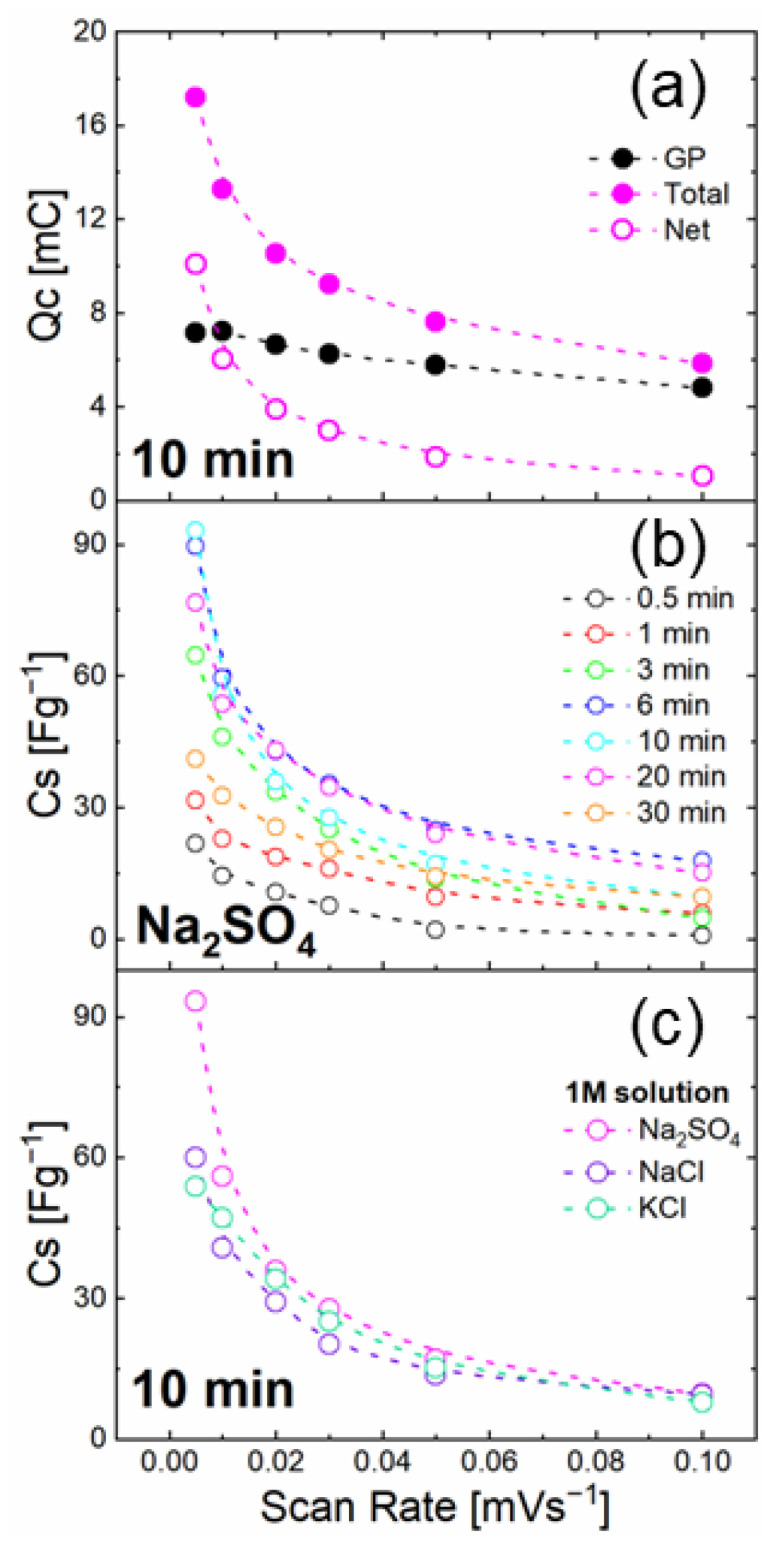
(**a**) Stored charge in GP (black symbols), 10 min ZnO NSs on GP (total, full magenta symbols) and their difference (net) (open magenta symbols); (**b**) specific capacitances extracted from CV for 0.5, 1, 2, 3, 6, 10, 20, and 30 min ZnO NSs in Na_2_SO_4_; and (**c**) specific capacitances of 10 min ZnO in NaCl, KCl, and Na_2_SO_4_ (purple, green, and magenta symbols respectively).

Figure 4a represents the stored charge from the CV as a function of the scan rate for GP and NSs on GP (total). The storage capacity for NSs (net) was extracted as the difference between these two. It should be noted that such a net storage capacity underestimates the ZnO NSs performances, as a non-negligible surface of the GP is covered by ZnO NSs, reducing the effective GP contribution to the charge storage in ZnO NSs-covered samples. The stored charge trend in the GP confirmed the EDLC mechanism due to its weak dependence on the scan rate (black spheres in Figure 4a). Instead, a strong dependence on the scan rate emerged for ZnO NS on GP (closed purples), with a stored charge of 17.2 mC at 5 ${\mathrm{mV}\;s}^{-1}$, evidencing that ZnO NSs act as energy storage materials with a different mechanism in comparison to that of GP. In fact, by plotting the net stored charge in ZnO NSs (open magenta spheres), a clear decrease was observed with the scan rate, decreasing the stored charge at very high scan rates almost to zero.

In order to extract an effective ZnO NSs specific capacitance, we took into account the net stored charge. Figure 4b shows these effective C_s_ for all the growth times. In all the cases, a marked dependence on the scan rate was observed, as for the 10 min growth time discussed above. Indeed, the growth time significantly affected the C_s_, with 6 and 10 min grown ZnO NSs exhibiting the largest values.

In order to study the effect of different electrolytes still at neutral pH, Figure 4c shows the effective C_s_ of the 10 min NSs as a function of the scan rate in 1 M ${\mathrm{Na}}_{2}{\mathrm{SO}}_{4}$ (magenta curve), 1 M KCl (green curve), and 1M NaCl (purple curve). While NaCl and KCl showed very similar results, ${\mathrm{Na}}_{2}{\mathrm{SO}}_{4}$ evidenced a larger specific capacitance at a lower scan rate.

Now, few considerations can be made in order to model the charge storage mechanism in ZnO NSs. By applying a positive voltage to WE, negative charges in the solution were attracted. $\mathrm{KCl},\;\mathrm{NaCl},{\;\mathrm{and}\;\mathrm{Na}}_{2}{\mathrm{SO}}_{4}$ dissociate as follows:

$$\mathrm{NaCl}\to {\;\mathrm{Na}}^{+}+{\mathrm{Cl}}^{-}$$

$$\mathrm{KCl}\;\to {\;K}^{+}+{\;\mathrm{Cl}}^{-}$$

$${\mathrm{Na}}_{2}{\mathrm{SO}}_{4}\to 2{\mathrm{Na}}^{+}+{\;\mathrm{SO}}_{4}^{2-}$$

Among negative ions, ${\;\mathrm{SO}}_{4}^{2-}$ is larger and slower, so it requires a longer time to reach the WE surface (also taking into account the solvation shells) [46]. Hence, at low scan rates, the doubly charged sulfate ion allows a larger storage capacity since its lower diffusion plays a minor role with respect to chloride ions.

For a better understanding of the storage mechanism involved, the Dunn model was applied to the 10 min NSs CV curve in 1 M ${\mathrm{Na}}_{2}{\mathrm{SO}}_{4}$. The total charge stored can be split into three components: a faradic contribution from ion insertion; a faradic contribution from charge-transfer processes with surface atoms, referred to as pseudocapacitance; and a non-faradic contribution from the double-layer effect [47]. At nanoscale dimensions, both double-layer charging and pseudocapacitance can be substantially due to the high surface area.

These effects can be calculated by examining the cyclic voltammetry data at various scan rates according to:

$$I=a\upsilon^{b}$$

where the measured current *I* obeys a power–law relationship with the scan rate *υ*, and *a* and *b* are adjustable parameters. Specifically, *b* is determined from the log*I* vs. log*υ* plot slope. There are two well-defined conditions since only diffusive or surface-limited phenomena can be present (*b* = 0.5 or 1, respectively) [47,48].

In Figure S5, the extrapolated *b* values for the 10 min NSs in 1 M ${\mathrm{Na}}_{2}{\mathrm{SO}}_{4}$ (magenta symbols) at different scan rates as a function of the potential values are presented. As previously explained, there were no peaks in the CV curves, so *b* never reached a value of 0.5, but swung between 0.75 and 0.9, indicating a dominant capacitive process with some pseudocapacitive contribution. The surface defects previously discussed in the PL analyses may play a fundamental role in WE surface charge-transfer phenomena.

**Figure 5:**
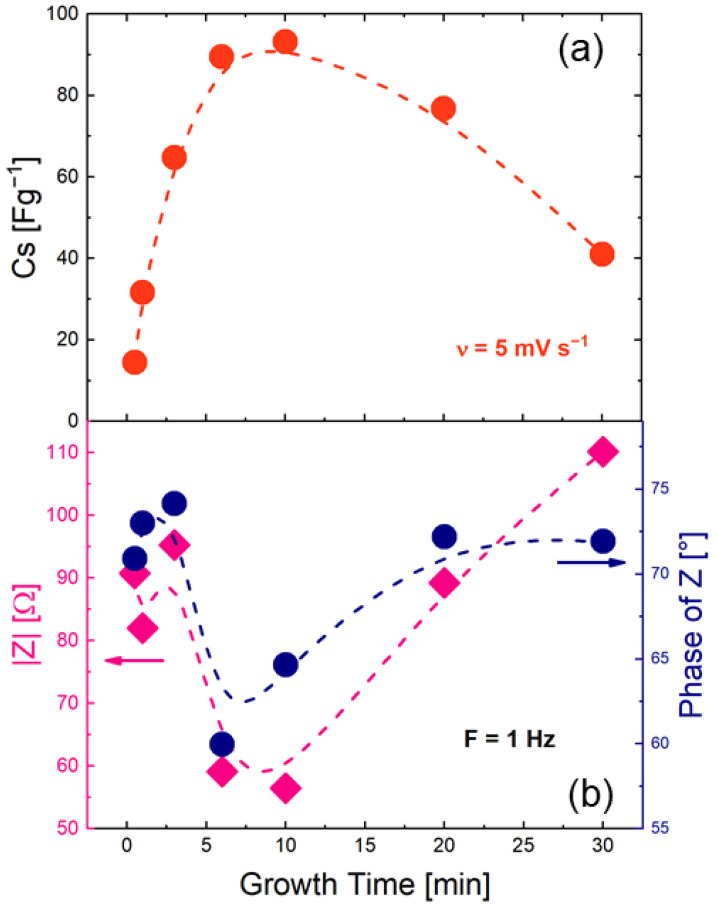
(**a**) C_s_ trend from CV curves acquired at 5 ${\mathrm{mV}\;s}^{-1}$ for all growth times analyzed and (**b**) impedance modulus and phase angle amplitude (magenta and blue symbols, respectively) trends (F = 1 Hz) as a function of growth time.

To determine what happens at the electrode–electrolyte interface, EIS analysis was performed at a potential of 0.3 V versus SCE (see Bode plot in Figure S6) and compared with specific capacitance values. Figure 5a shows C_s_ (*υ* = 5 ${\mathrm{mV}\;s}^{-1}$) as a function of growth time. The C_s_ values exhibited a clear bell-shaped trend. Figure 5b shows the impedance modulus and phases angle amplitudes at 1 Hz as a function of growth time. Focusing on the impedance modulus (blue), a funnel-shaped trend can be recognized. The 10 min growth point had the lowest impedance value (56 Ω), which is specular with the highest value of C_s_ (94 ${F\;g}^{-1}$) found for the same sample. The impedance module is inversely related to capacitance [49]. Hence, these two quantities being inversely proportional, lower |Z| values mean higher capacitance values. It is unequivocal that the impedance modulus trend (magenta curve, Figure 5b) is the C_s_ bell trend's mirror image (red curve, Figure 5a).

**Figure 6:**
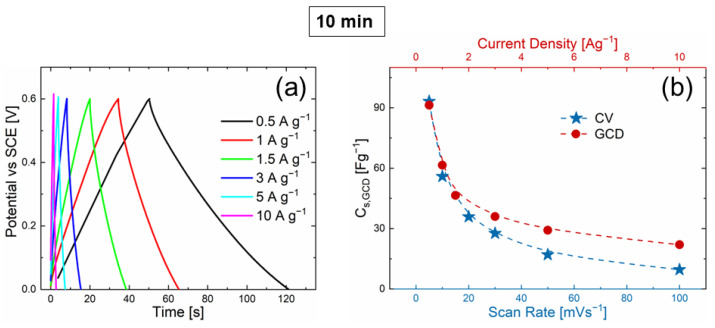
(**a**) GCD curves (0.5 A g^−1^ black, 1 A g^−1^ red, 1.5 A g^−1^ green, 3 A g^−1^ blue, 5 A g^−1^ light blue, and 10 A g^−1^ magenta lines) and (**b**) specific capacitance obtained by GCD (red dashed line and circles) and CV (light blue dashed line and stars) curves, trends of 10 min NS.

To counterproof the ZnO NSs charge storage process in real conditions, GCD curves were recorded. Figure 6a shows the GCD curves obtained at different current densities (from 0.5 to 10 ${A\;g}^{-1}$) in the same voltage range as that in the CV analyses. As expected, the NSs discharge time decreased with the increase in current density. The charge and discharge curves are symmetric, which indicates high reversibility, high columbic efficiency, and poor energy loss during the charge and discharge process. C_s,GCD_ can be calculated from the GCD as follows [45]:

$$C_{s,\mathrm{GCD}}=\frac{It_{s}}{m\Delta V}$$

where $t_{s}$ is the discharge time (s), I is the applied current (mA), ΔV is the voltage window (V), and m is the active ZnO NSs mass (mg). Figure 6b shows C_s,GCD_ as a function of the scan rate (CV, blue curve) and as a function of current density (GCD, red curve). The C_s,GCD_ trend matched well with the values of the CV analyses, hence confirming again that all the data were consistent.

## 4. Conclusions

To conclude, we report novel ZnO nanostructures with a six-point star shape that have a good energy storage ability in neutral pH conditions. A large amount of ZnO/ZnOHF nanostars, highly ordered within the plane, were obtained via the CBD method. By varying the CBD duration, star arm elongation was observed, while annealing up to 300 °C induced a material purification towards the ZnO phase with concomitant surface oxygen vacancy defect removal. Energy storage was tested by covering a graphene paper substrate with ZnO nanostars and performing the electrochemical measurements. The ZnO nanostars showed a marked pseudocapacitive behavior at all the growth times, both as prepared and after annealing. The best performance was reached for the 10 min growth time without thermal treatments because of the presence of surface defects and low electrochemical impedance values. A careful measurement of the ZnO nanostars’ net capacitance determined a maximum value of 94 ${F\;g}^{-1}$. The specific capacitance evaluation from the GCD was highly consistent with the values calculated from the CV analyses. All the results suggest that ZnO-based NSs can find promising application in efficient energy storage devices owing to the synergy between the double-layer capacitance and the pseudocapacitive effect.

## Data Availability

The data presented in this study are available on request from the corresponding author.

## Supplementary Materials

The following supporting information can be downloaded at: https://www.mdpi.com/article/10.3390/nano12152588/s1, Figure S1. (a) High magnification SEM of an NS center and (b) an NS tip. Figure S2. SEM image of AnnNSs. Figure S3. EDX spectrum of NSs taken in NSs grown for 10 min. Figure S4. SEM micrograph of (a) single grown NSs and (b–d) related SEM-EDX mapping for O, F, and Zn elements, respectively. EDX maps were acquired setting the operating voltage as 20 kV and a working distance of 8.5 mm. Figure S5. Extrapolated b values (magenta circles) for 10 min NSs in 1 M Na_2_SO_4_ at different scan rates as a function of potential values. Figure S6. Bode plot from the EIS analyses acquired at 0.3 V: (a) impedance modulus and (b) phase angle amplitudes for all growth times analyzed. Data for the GP substrate are also reported (GP grey, 0.5 min black, 1 min red, 3 min green, 6 min blue, 10 min light blue, 20 min magenta, and 30 min Bordeaux lines and circles).
