# Supplementary material for: Low-Cost, High-Yield ZnO Nanostars Synthesis for Pseudocapacitor Applications
Source: Nanomaterials (Basel). 2022 Jul 28;12(15):2588. doi: 10.3390/nano12152588 (PMC9370347; doi:10.3390/nano12152588)
Supplement: Supplementary file 1 [file nanomaterials-12-02588-s001.zip › nanomaterials-1792902-supplementary.pdf]

Supplementary Materials

Low-Cost, High-Yield ZnO Nanostars Synthesis for Pseudocapacitor Applications

Gisella Maria Di Mari [1,2], Giacometta Mineo [1,2], Giorgia Franzò [2], Salvatore Mirabella [1,2,*], Elena Bruno [1,2] and Vincenzina Strano [2]

[1] Dipartimento di Fisica e Astronomia "Ettore Majorana", Università degli Studi di Catania, via S. Sofia 64, 95123 Catania, Italy; gisella.dimari@dfa.unict.it (G.M.D.M.); giacometta.mineo@dfa.unict.it (G.M.); elena.bruno@dfa.unict.it (E.B.)

[2] Consiglio Nazionale delle Ricerche, Istituto per la Microelettronica e i Microsistemi (CNR-IMM), Università degli Studi di Catania, via S. Sofia 64, 95123 Catania, Italy; giorgia.franzo@ct.infn.it (G.F.); vincenzina.strano@ct.infn.it (V.S.)

* Correspondence: salvo.mirabella@dfa.unict.it

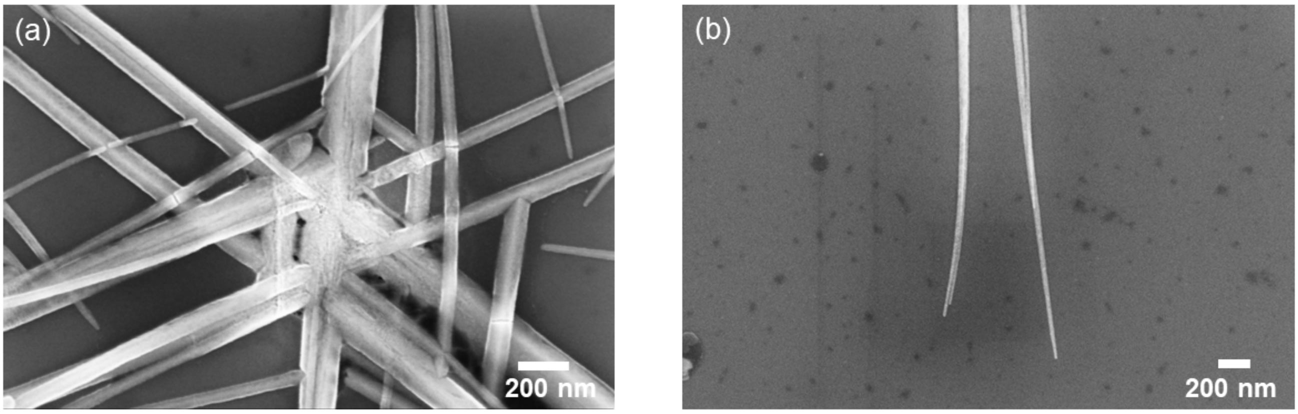

Figure S1. (**a**) High magnification SEM of an NS center and (**b**) an NS tip.

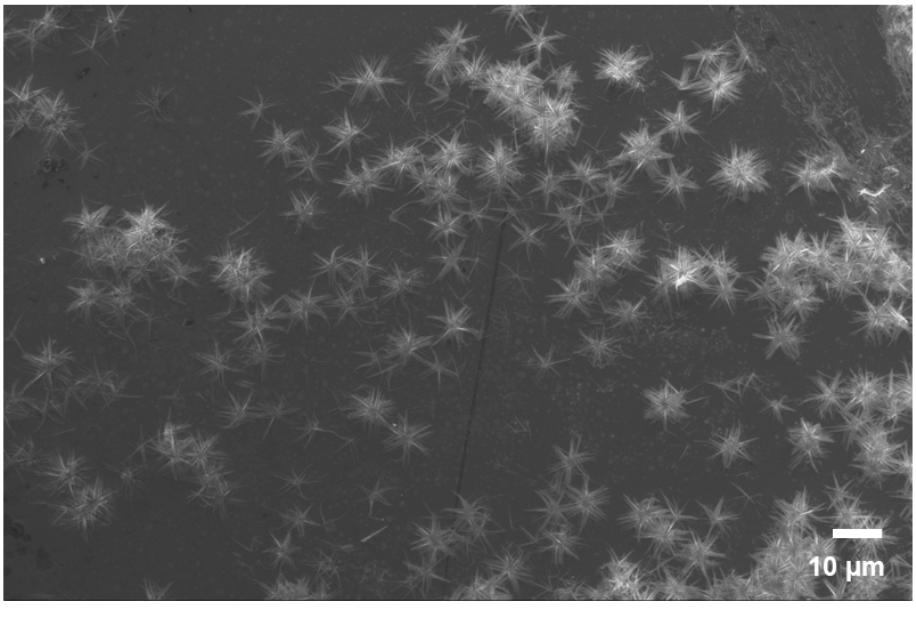

Figure S2. SEM image of AnnNSs.

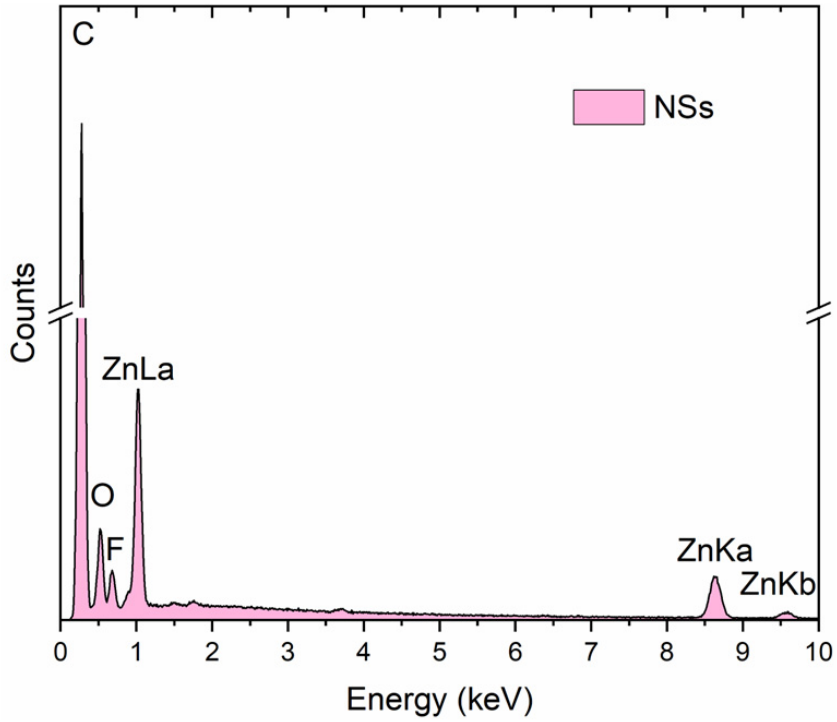

Figure S3. EDX spectrum taken in NSs grown for 10 min.

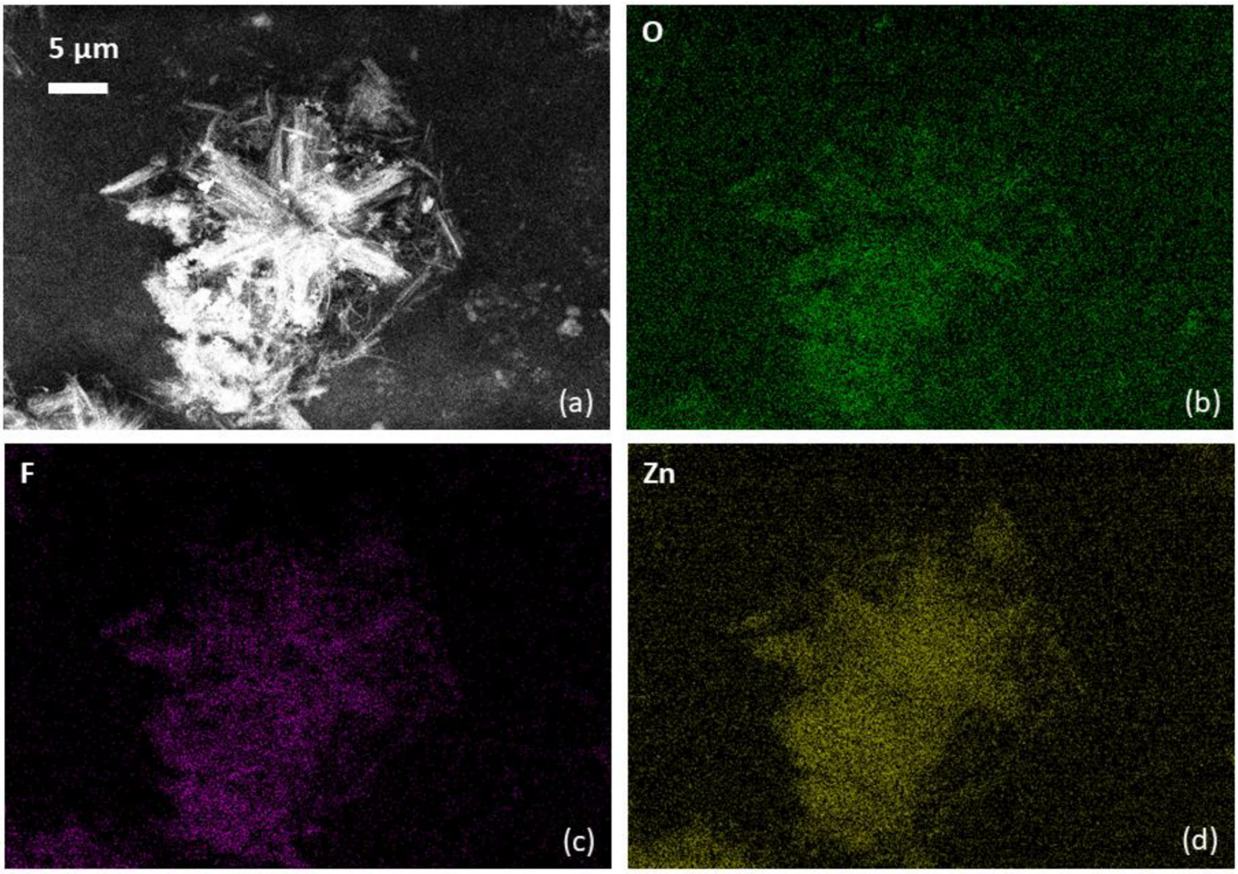

Figure S4. SEM micrograph of (**a**) single grown NS and (**b**–**d**) related SEM-EDX mapping for O, F and Zn elements respectively. EDX maps were acquired setting the operating voltage as 20 kV and a working distance of 8.5 mm.

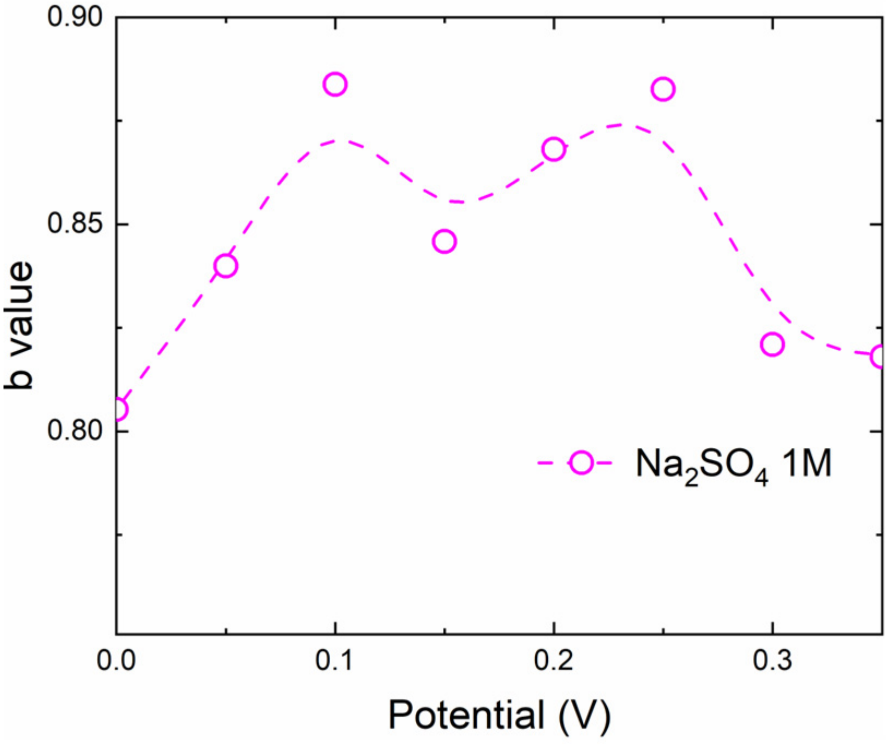

Figure S5. Extrapolated b values for 10 min NSs in 1 M Na_2SO_4 at different scan rates as a function of potential values.

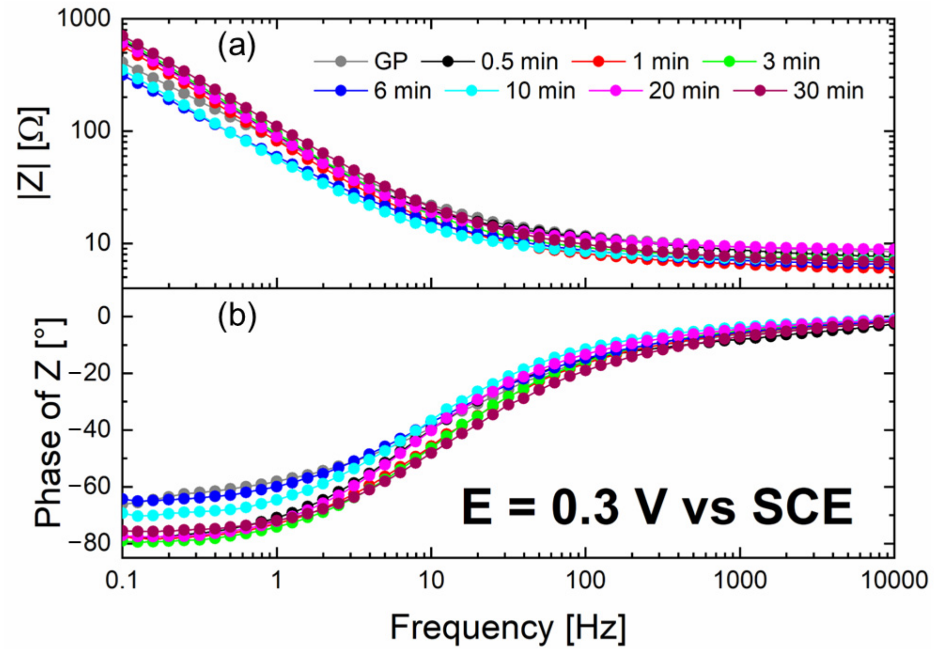

Figure S6. Bode Plot from the EIS analyses acquired at 0.3 V: (**a**) impedance modulus and (**b**) phase angle amplitudes for all growth times analyzed. Data for the GP substrate are also reported (GP grey, 0.5 min black, 1 min red, 3 min green, 6 min blue, 10 min light blue, 20 min magenta, and 30 min Bordeaux lines and circles).
